# Multi-Layer Perception model with Elastic Grey Wolf Optimization to predict student achievement

**DOI:** 10.1371/journal.pone.0276943

**Published:** 2022-12-30

**Authors:** Yinqiu Song, Xianqiu Meng, Jianhua Jiang

**Affiliations:** 1 College of Foreign Languages, Wuzhou University, Wuzhou, P. R. China; 2 School of Management Science and Information Engineering, Jilin University of Finance and Economics, Changchun, P. R. China; Suleyman Demirel University, TURKEY

## Abstract

This study proposes a Grey Wolf Optimization (GWO) variant named Elastic Grey Wolf Optimization algorithm (EGWO) with shrinking, resilient surrounding, and weighted candidate mechanisms. Then, the proposed EGWO is used to optimize the weights and biases of Multi-Layer Perception (MLP), and the EGWO-MLP model for predicting student achievement is thus obtained. The training and verification of the EGWO-MLP prediction model are conducted based on the thirty attributes from the University of California (UCI) Machine Learning Repository dataset’s student performance dataset, including family features and personal characteristics. For the Mathematics (Mat.) subject achievement prediction, the EGWO-MLP model outperforms one model’s prediction accuracy, and the standard deviation possesses the stable ability to predict student achievement. And for the Portuguese (Por.) subject, the EGWO-MLP outperforms three models’ Mathematics (Mat.) subject achievement prediction through the training process and takes first place through the testing process. The results show that the EGWO-MLP model has made fewer test errors, indicating that EGWO can effectively feedback weights and biases due to the strong exploration and local stagnation avoidance. And the EGWO-MLP model is feasible for predicting student achievement. The study can provide reference for improving school teaching programs and enhancing teachers’ teaching quality and students’ learning effect.

## Introduction

Education refers to school education organized by particular organizations from a narrow view. And from a broad perspective, it relates to social practice activities that affect people’s physical and mental development [1]. According to school conditions and professional titles, it aims to educate and cultivate cognitive development in a planned and organized way, teach people with existing experience and knowledge [2, 3], explain various phenomena, problems, or behavior [4], and improve their practical ability. It is fundamental to recognize and treat things with people’s relatively mature or rational thinking.

In contemporary society, with the rapid development of information technology, the deepening of education and teaching reform has also been deeply affected [5]. Especially in the era of big data, various data mining methods are applied to the education industry, which provides a new idea for finding better educational and teaching methods [6, 7]. Due to the continuous progress of education, more and more advanced techniques are constantly used by the education industry to help decision-makers carry out educational analysis [8] and provide better educational effects by continuously developing the existing education system. Student performance and achievement prediction can urge students to strengthen learning efficiency [9, 10], encourage teachers to boost teaching quality [11, 12], and better improve teaching to achieve the best effect [13]. Student achievement is not only an important indicator to measure talent training level but also an essential part of big educational data [14]. Accordingly, student achievement prediction is the leading research direction of scholars.

Student achievement is characterized by a relatively unified data type, a large data volume, and relatively easy access. It has become a research hotspot of teaching reform to conduct in-depth mining and analysis of student achievement from multiple angles according to appropriate data mining technology [14, 15]. The above descriptions demonstrate that student education is a non-linear, higher-dimension problem [16]. Traditional technology is challenging to solve high-dimensional and complex problems, and obtaining new methods or theories to guide teaching is a significant task [17].

Artificial neural networks (ANNs) are an algorithmic mathematical model that imitates the behavior characteristic of animal neural networks for distributed parallel information processing. Depending on the complex system, this network achieves the purpose of processing information by adjusting the inter-connected relationship between plenty of internal nodes obtaining the ability of self-learning and self-adapting [18, 19]. ANNs are widely used in various complex practices, such as predicting performance, modeling, and grouping of students according to their personal characteristics, providing personalized learning support for students, and so on [20]. Multi-Layer Perceptron (MLP) is the most straightforward neural network, which can contain one or more hidden layers, and has been applied to many fields [21, 22].

The swarm intelligence optimization algorithm is inspired by the behavior of group communication and predation in nature. Experts and scholars have proposed many novel and efficient swarm intelligence optimization algorithms, such as Ebola Optimization Search Algorithm (EOSA) [23], multi-objective multi-objective (MOAVOA) [24] and so on. The above algorithms are of good parallelism and autonomous exploration, provide new ideas and methods for solving complex problems and have become the focus of increasing attention among researchers. It has been widely for solving practical problems [25, 26] and for optimizing the weights and biases of the MLP [27]. The Grey wolf optimization (GWO) algorithm is a kind of swarm intelligence optimization algorithm [28]. Based on the wolf hierarchy, this algorithm simulates wolves’ behaviors of surrounding, following, and hunting prey. It has a better ability to solve high-dimensional complex problems [29, 30]. Since it was put forward, it has attracted the attention of many scholars and applied to various fields. In recent years, combining neural networks to solve practical problems has had a high interional profile [31, 32].

Following No Free Lunch (NFL) thoery, no algorithm can effectively solve all problems [33]. When solving different problems, the algorithm will encounter such unfavorable situations as local stagnation, premature convergence, unbalanced exploration and exploitation, etc. Therefore, to avoid the above problems, this paper proposes a GWO variant called Elastic Grey Wolf Optimization algorithm (EGWO) to train Multi-Layer Perceptron and optimize weights and biases.

**Fig 1:**
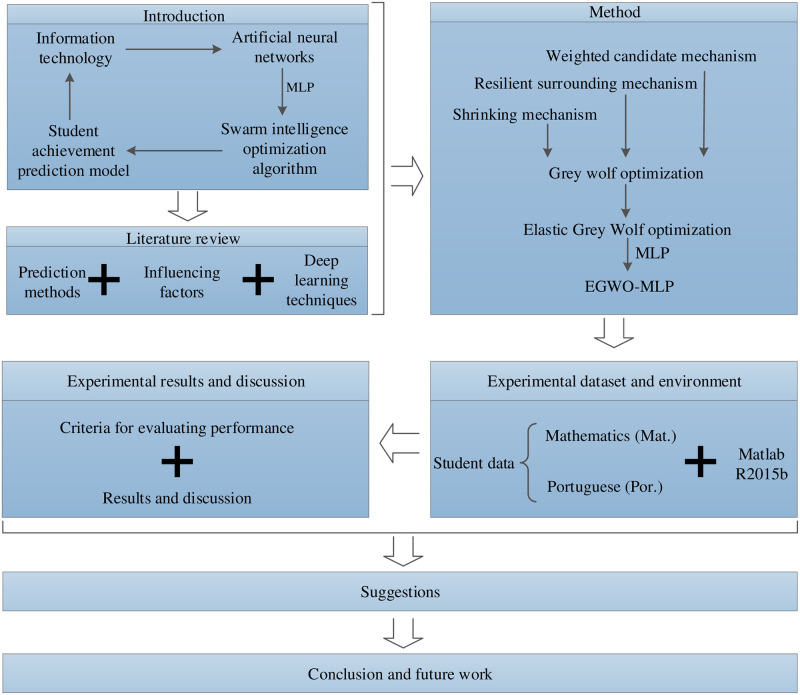
The full paper overall structure.

The architectural idea of this paper is shown in Fig 1.

## Literature review

Many scholars have conducted studies on student performance to make full use of educational big data resources and have promoted the peaceful development of teaching reform.

Scholars apply various methods to predict student performance. Woo et al. study the relationship between indoor conditions and students’ classroom performance and use a hierarchical multiple regression model to determine the crucial predictors of student performance [34]. Based on different central dimensions, Vignery predicts student performance through Principle Component Analysis (PCA), Exponential Random Graph Models (ERGM), agglomerative hierarchical clustering, and multilevel modeling. The experimental results show that geodesic k-path and closeness centrality positively impact Grade Point Agerage (GPA) [35]. To help teachers offer help in time to improve students’ academic performance, Khan et al. classify student performance before the beginning of the class and propose a novel classification method, which can provide an additional confidence measure and improve the acceptability of prediction [36]. Different principal leaders impact student performance as a significant force in the school. Wu et al. conduct a Multivariate meta-meta-analysis to investigate the relationship between the central leadership and student achievement [37]. Based on the DEA model and Bootstrap method, Masci et al. measure the impact of school (district) size, management practice, and principal leaders’ characteristics on student groups through reading and mathematics standardized test scores [38]. The experimental results show that the composition of school subjects mainly affects students’ reading efficiency, and management practice mainly affects students’ efficiency of mathematics.

Scholars choose various characteristics to predict student performance. Many higher education institutions try to understand student factors to improve the quality of education. Students’ semantic trajectory is tested by dynamic time distortion, hierarchical clustering, and variance analysis. The experimental results show that semantic trajectory is a relevant factor affecting student performance [39]. Silva et al. analyze family characteristics, values, beliefs, expectations and family support, self-efficacy, goal progress, and academic achievement [40]. The results show that families affect academic performance through academic self-efficacy and views on the progress of educational goals. However, the information provided by self-efficacy has less impact but is more related to the support of material resources. Sarfraz et al. study the factors affecting the performance of business school students during the COVID-19 pandemic: assessing students’ views and preferences, the impact of blended learning (BL) setting on students’ academic performance, and studying the relationship between unified theory of acceptance and use of the technology of UTAUT and students’ academic performance [41]. Javadizadeh et al. study the impact of class structure, teaching style, and class environment on students’ classroom realization, and draw lessons from the self-determination theory to assume the relationship between SCARF (status, certainty, autonomy, relatedness, fairness) elements, students’ internal motivation, and classroom performance [42]. This study has important guiding significance for improving students’ enthusiasm.

Some scholars also choose deep learning techniques to predict student grades, which can be summarized as follows. Rivas et al., to understand the influencing factors of college students, determine the critical factors of student performance through the number of visits to available resources, based on the tree model and different types of ANNs [43]. Li et al. propose a Multi-View Hypergraph Neural Network (MVHGNN) for predicting students’ academic performance, which uses hypergraphs to construct high-order relations among students. And a Cascade Attention Transformer (CAT) module is introduced to mine the weight of different behaviors by the self-attention mechanism. The experimental results demonstrate that the MVHGNN method outperforms the state-of-the-art ones evaluated on real campus student behavioral datasets [44]. Bertolini et al. utilize bootstrapping to examine performance variability among five data mining methods (DMMs) and four filter preprocessing feature selection techniques for forecasting course grades for 3225 students enrolled in an undergraduate biology class [45]. Wu et al. propose a novel knowledge tracing model based on an exercise session graph, named session graph-based knowledge tracing (SGKT). The session graph models the students’ answering process. And a relationship graph models the relationship between exercises and skills. The experimental results demonstrate the model can outperform some existing baseline methods conducted on three publicly available datasets [46]. Pallathadka et al. analyze the ability of machine learning, such as Naive Bayes, ID3, C4.5, and SVM to predict the students’ performance in future tests. And the above methods are evaluated by criteria like accuracy and error rate by the UCI machinery student performance data set [47]. Tomasevic et al. accomplish the task of student exam performance prediction, i.e., discovering students at a “high risk” of dropping out from the course by providing a comprehensive analysis and comparison of state-of-the-art supervised machine learning techniques and predicting their future achievements, such as instance, final exam scores [48].

Although scholars have adopted different methods to study the factors affecting student performance and achievement [49–51], and to predict final performance [52, 53]. There are few studies conducted on the impact of students’ characteristics and family factors on their achievement [54, 55].

## Method

### Ethics statement

In this thesis, the standard University of California (UCI) Machine Learning Repository dataset (https://archive.ics.uci.edu/ml/datasets/Student+Performance) is selected in the simulation experiment. The standard UCI dataset is usually used as a general dataset and often appears in most papers or studies, and the original data are provided on the official website. The Student Performance Data Set introduced in the experimental dataset and environment section are obtained from the official website, which is from two Portuguese secondary schools collected through reports and questionnaires on the performance of students in secondary education. It does not involve human participants, human specimens or tissue, vertebrate animals or cephalopods, vertebrate embryos or tissues, and field research.

### Grey Wolf Optimizer

Grey Wolf Optimizer (GWO) is a swarm intelligence algorithm that mimics the hunting behavior of wolves [28]. The superior performance of this algorithm benefits from the wolf herd hierarchy mechanism. Among wolves, *α*, *β*, and *δ* wolves are the three primary wolves. The rest are named *ω* wolves, in the lowest class, to attack the prey. GWO algorithm incorporates two primary operations: (1) surrounding the prey and (2) hunting the prey. The whole process of the algorithm is as shown in the Algorithm 1.

**Operation 1: Surrounding the prey**. Eqs (1) and (2) express the surrounding mechanism.

$$\begin{array}{c} D=|C*x_{p}\left(t\right)-x\left(t\right)| \end{array}$$ (1)

$$\begin{array}{c} x\left(t+1\right)=x_{p}\left(t\right)-A*D \end{array}$$ (2)

where *t* is the current number of iteration; *x*_*p*_(*t*) is the location of the prey (equivalent to *α*, *β*, *δ* and *ω*), *x*(*t*) and *x*(*t* + 1) are wolf locations of the *t*_*th*_ and (*t* + 1)_*th*_ iteration. *D* is the distance between the prey and the wolf. *A* and *C* are calculated by Eqs (3) and (4).

$$\begin{array}{c} A=2*a*r_{1}-a \end{array}$$ (3)

$$\begin{array}{c} C=2*r_{2} \end{array}$$ (4)

where *a* is reduced linearly from 2 to 0 along with the iterations, and *r*_1_ and *r*_2_ are random values in the range of [0, 1].

**Operation 2: Hunting the prey**. The *α* wolf leads the whole process. The final position is to update Eqs (5) to (11) at any position in the circle. The *ω* wolves randomly update around the prey, and *α*, *β*, and *δ* wolves evaluate the prey location.

$$\begin{array}{c} D_{\alpha }=|C_{\alpha }*x_{\alpha }-x_{i}| \end{array}$$ (5)

$$\begin{array}{c} D_{\beta }=|C_{\beta }*x_{\beta }-x_{i}| \end{array}$$ (6)

$$\begin{array}{c} D_{\delta }=|C_{\delta }*x_{\delta }-x_{i}| \end{array}$$ (7)

$$\begin{array}{c} x_{1}=x_{\alpha }-A_{\alpha }*\left(D_{\alpha }\right) \end{array}$$ (8)

$$\begin{array}{c} x_{2}=x_{\beta }-A_{\beta }*\left(D_{\beta }\right) \end{array}$$ (9)

$$\begin{array}{c} x_{3}=x_{\delta }-A_{\delta }*\left(D_{\delta }\right) \end{array}$$ (10)

$$\begin{array}{c} x\left(t+1\right)=\frac{x_{1}+x_{2}+x_{3}}{3} \end{array}$$ (11)

**Algorithm 1**: The basic Grey Wolf Optimization Algorithm

**Step 1**: Initialize the grey wolf population: *X*_*i*_ (i = 1,2,…,n);

**Step 2**: Initialize the parameters: *a*, *A* and *C*;

**Step 3**: Calculate the fitness value of each wolf:

*X*_*α*_ ← *best*(*f*(*X*_*i*_)); *X*_*β*_ ← *second* − *best*(*f*(*X*_*i*_)); *X*_*δ*_ ← *third* − *best*(*f*(*X*_*i*_));

**Step 4**: **While t** < *t*_*max*_

 **For each wolf**

  Upate the current position based on the Eqs (1) to (11);

 **End For**

 **End While**

**Step 5**: Return *X*_*α*_.

### The variant of the GWO

Since the GWO was proposed, it has attracted the attention of many scholars. In the past two years (2021 and 2022), many scholars have proposed a variety of variants, some of which are as follows:

- **Ensemble Grey Wolf Optimizer (EGWO)**: Yu et al. propose a variant Ensemble GWO (EGWO) with two strategies to boost the performance of GWO, which is validated by the IEEE CEC 2019 and image segmentation in the real world. The results show that the proposed EGWO algorithm is reliable and effective [56].
- **Random Walk Grey Wolf Optimizer (RWGWO)**: Deep et al. propose a Random Walk Grey Wolf Optimizer based on the dispersion factor (RWGWO) approach to the feature selection problem, and it examines eighteen different chronic disease data. The experimental results show the RWGWO is an effective GWO variant [57].
- **Diversity enhanced Strategy based Grey Wolf Optimizer (DSGWO)**: To solve the poor population diversity and global search capabilities, Jiang et al. propose Diversity enhanced Strategy based Grey Wolf Optimizer (DSGWO), which combines group-stage competition and the exploration-exploitation balance mechanisms to improve the performance of the GWO algorithm. The DSGWO is validated by IEEE CEC 2014 and two engineering design problems, and the results prove that the DSGWO has a strong exploration and exploitation ability [58].
- **Adult-Pup Teaching–Learning based Interactive Grey Wolf Optimization (AP-TLB-IGWO)**: Banerjee et al. propose adult-pup teaching–learning based interactive grey wolf optimization (AP-TLB-IGWO) algorithm to mitigate these challenges and for the better representation of the existing system. The performance of the AP-TLB-IGWO is tested by the IEEE CEC 2014 and CEC 2017, which provide significantly promising results in comparison with current techniques [59].
- **Hybrid grey wolf optimization (HGWO)**: Hoballah et al. propose a variant of Hybrid grey wolf optimization (HGWO) with two internal loops based on particle swarm optimization (PSO) and genetic algorithm (GA) techniques. And the HGWO is conducted on a 66-bus three-area test system for cost minimization following the outage of the biggest generator in each area [60].
- **Randomized Balanced Grey Wolf Optimizer (RBGWO)**: Adhikary et al. propose a new variant of GWO termed Randomized Balanced Grey Wolf Optimizer (RBGWO), which outperforms some meta-heuristic algorithms. The proposed algorithm is applied to constrained and unconstrained real life problems. The results produced by the proposed variant are of better quality compared to those of others [61].
- **Mutation-driven Modified Grey wolf (MDM-GWO)**: Singh et al. propose a new variant of the GWO called Mutation-driven Modified Grey wolf (MDM-GWO). The variant’s performance is tested by 23 well-known standard benchmark problems and four real world engineering design problems. The numerical results, statistical tests, convergence and diversity curves, and comparisons among several algorithms show the superiority of the proposed MDM-GWO [62].
- **Binary Grey Wolf Optimization (BGWO)**: To solve the Virtual Network Function (VNF), Shahjalal et al. propose an artificial intelligence (AI) driven meta-heuristic Binary Grey Wolf Optimization (BGWO) algorithm for VNF deployment. The results show the method can minimize VNF deployment costs and maximize users’ QoE [63].
- **Gaze cues learning-based grey wolf optimizer (GGWO)**: Nadimi-Shahraki et al. propose Gaze cues learning-based grey wolf optimizer (GGWO) with the two mechanisms of the neighbor gaze cues learning (NGCL) and random gaze cues learning (RGCL) inspired by the gaze cueing behavior in wolves. Four real engineering design problems and two optimal power flow (OPF) problems for the IEEE 30-bus and IEEE 118-bus are optimized to verify the applicability of the GGWO in practice. And the results show that the GGWO algorithm is able to provide competitive and superior results to the compared algorithms [64].
- **Improved grey wolf optimizer (IGWO)**: Wang et al. propose an improved grey wolf optimizer (IGWO) to optimize the model. The experimental results show the superiority of the proposed method compared with other algorithms for solving WSISIP [65].
- **Advanced Grey Wolf Optimization algorithm (AGWO)**: Meng et al. propose a variant named Advanced Grey Wolf Optimization algorithm (AGWO) with elastic, circling, and attacking mechanisms. The variant optimizes the weights and biases of the MLP. Seven classification and three function approximation datasets investigate the performance of the AGWO, and the results show that the AGWO is superior to some other heuristic algorithms in local optimum avoidance and computational accuracy [19].

### Multi-Layer Perceptron (MLP)

**Fig 2:**
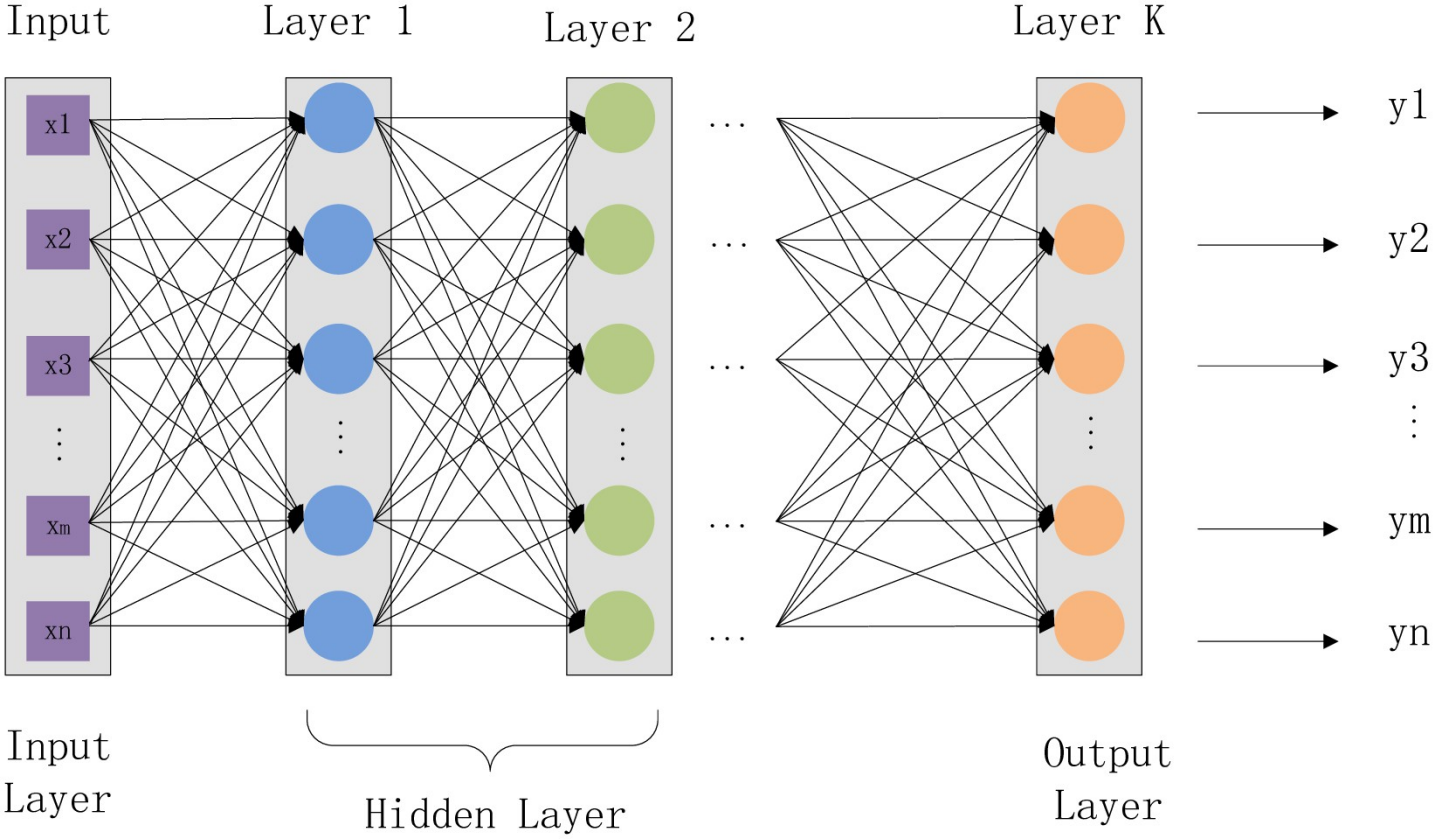
A simple Multi-Layer Perceptron (MLP).

Multi-Layer Perceptron (MLP) is an artificial neural network with a forward structure, which maps a set of input vectors to a group of output vectors [66, 67]. MLP (as shown in Fig 2) can be regarded as a directed graph composed of multiple node layers, and each layer connects to the next layer. Each node is a neuron (or processing unit) with a non-linear activation function. The basic structure of multi-layer perception consists of three layers: the first input layer, the middle hidden layer, and the last output layer. The input elements and weights results feed to the summation node with neuron bias. The primary calculation process is as follows:

(1) The weighted sum of the inputs can be calculated by Eq (12).

$$\begin{array}{c} S_{j}=\sum_{i=1}^{n}\left(W_{ij}X_{i}\right)-\theta_{j},j=1,2,....h \end{array}$$ (12)

where *n* is the number of the input nodes, *W*_*ij*_ indicates the weight linking the *i*_*th*_ input layer node and the *j*_*th*_ hidden layer node, and *X*_*i*_ presents the *i*_*th*_ input.

(2) The hidden nodes output can be calculated by Eq (13).

$$\begin{array}{c} S_{j}=sigmoid\left(s_{j}\right)=\frac{1}{\left(1+exp\left(-s_{j}\right)\right)},j=1,2,....h \end{array}$$ (13)

(3) The hidden node and final output are calculated by Eqs (14) and (15).

$$\begin{array}{c} o_{k}=\sum_{j=1}^{h}\left(w_{jk}S_{j}\right)-\theta_{k}^{\prime },k=1,2....m \end{array}$$ (14)

$$\begin{array}{c} O_{k}=sigmoid\left(o_{k}\right)=\frac{1}{\left(1+exp\left(-o_{k}\right)\right)},k=1,2,....m \end{array}$$ (15)

where *w*_*jk*_ is the weight connecting the *j*_*th*_ hidden node with the *k*_*th*_ output node, *θ*_*j*_ and $\theta_{k}^{\prime }$ are the biases of the *j*_*th*_ hidden node and *k*_*th*_ output node.

### Elastic Grey Wolf Optimization algorithm (EGWO)

To improve the performance of the basic GWO, the shrinking, resilient surrounding, and weighted candidate mechanisms are introduced to remedy the local stagnation and premature convergence deficiencies of the proposed Elastic Grey Wolf Optimization algorithm (EGWO).

**Shrinking mechanism**. Cauchy distribution is a continuous probability distribution in which mathematical expectation does not exist. When the random variable satisfies its probability density function, the variable obeys the Cauchy distribution. To prevent the wolf position from falling into local stagnation, the parameters *A* and *C* are updated, inspired by Cauchy distribution, to propose a shrinking mechanism. The parameters *A* and *C* are computed by Eqs (3) and (4), and the random constant *r*_1_ and *r*_2_ are updated by Eqs (16) and (17). Parameter *a* can be calculated by Eq (18).

$$\begin{array}{c} r_{1}=tan\left(\left(rand-1/2\right)*\pi \right)) \end{array}$$ (16)

$$\begin{array}{c} r_{2}=tan\left(\left(rand-1/2\right)*\pi \right)) \end{array}$$ (17)

$$\begin{array}{c} a=2-l*{\left(\frac{2}{max_{iter}}\right)}^{2} \end{array}$$ (18)

**Resilient surrounding mechanism**. The core of the GWO algorithm is the movement and location of three *α*, *β*, and *δ* wolves. The original location updating strategy can not effectively solve high-dimensional and complex problems. It is difficult to find the best solution due to the local stagnation and premature convergence when solving the above complex real world problems. Therefore, introducing the resilient surrounding mechanism can overcome the above weakness. The position of *α*, *β*, and *δ* wolves can be updated by Eqs (19)–(22).

$$\begin{array}{c} D_{\alpha }=x_{\alpha }-x_{r}\;D_{\beta }=x_{r}-x_{\beta }\;D_{\delta }=x_{\delta }-x_{i} \end{array}$$ (19)

$$\begin{array}{c} x_{1}=x_{\alpha }+C_{\alpha }*\left(D_{\alpha }\right) \end{array}$$ (20)

$$\begin{array}{c} x_{2}=x_{\beta }+C_{\beta }*\left(D_{\beta }\right) \end{array}$$ (21)

$$\begin{array}{c} x_{3}=x_{\delta }+C_{\delta }*\left(D_{\delta }\right) \end{array}$$ (22)

**Weighted candidate mechanism**. In the basic GWO, the *α* wolf is used for hunting the prey, leading to local stagnation. Therefore, introducing the weighted candidate mechanism can avoid the above problem. First, the weighted coefficient can be computed by Eq (23) to adjust the step direction and length. Second, candidate wolves prepare to hunt the prey, and positions can be updated by Eqs (24) to (27). The whole process of the algorithm is as shown in Algorithm 2.

$$\begin{array}{c} w_{1}=A_{1}*C_{1}\;w_{2}=A_{2}*C_{2}\;w_{3}=A_{3}*C_{3} \end{array}$$ (23)

$$\begin{array}{c} CandPos\left(1,j\right)=\frac{\left(w_{2}*x_{1}+w_{1}*x_{2}\right)}{\left(w_{1}+w_{2}\right)} \end{array}$$ (24)

$$\begin{array}{c} CandPos\left(2,j\right)=\frac{\left(w_{3}*x_{1}+w_{1}*x_{3}\right)}{\left(w_{1}+w_{3}\right)} \end{array}$$ (25)

$$\begin{array}{c} CandPos\left(3,j\right)=\frac{\left(w_{3}*x_{2}+w_{2}*x_{3}\right)}{\left(w-2+w_{3}\right)} \end{array}$$ (26)

$$\begin{array}{c} CandPos\left(4,j\right)=\frac{\left(w_{3}*x_{1}+w_{2}*x_{2}+w_{1}*x_{3}\right)}{\left(w_{1}+w_{2}+w_{3}\right)} \end{array}$$ (27)

**Algorithm 2**: Elastic Grey Wolf Optimization algorithm (EGWO)

**Step 1**: Initialize population of *N* wolves: $W^{0}=w_{1}^{0},w_{2}^{0},...,w_{N}^{0}$;

**Step 2**: Evaluate population in the objective function;

**Step 3**: **While** (**t** < *t*_*max*_)

  Set top three wolves as *α*, *β* and *δ* wolves:

  *X*_*α*_ ← *best*(*f*(*X*_*i*_)); *X*_*β*_ ← *second* − *best*(*f*(*X*_*i*_)); *X*_*δ*_ ← *third* − *best*(*f*(*X*_*i*_));

**Step 4**: **For every wolf**

  Set *r*_1_ and *r*_2_ by Eqs (16) and (17);

  Initialize *a* according to the Eq (18);

  Update *X*_*α*_, *X*_*β*_ and *X*_*δ*_ according to Eqs (20)–(22);

  Update the weight *w*_1_ to *w*_3_ by Eq (23);

  Generate the candidate wolves by Eqs (24) to (27);

  Update the wolf population;

 **End For**

 t ← t+1

 **End While**

**Step 5**: Return *X*_*α*_.

### EGWO-MLP: Student achievement prediction model

**Fig 3:**
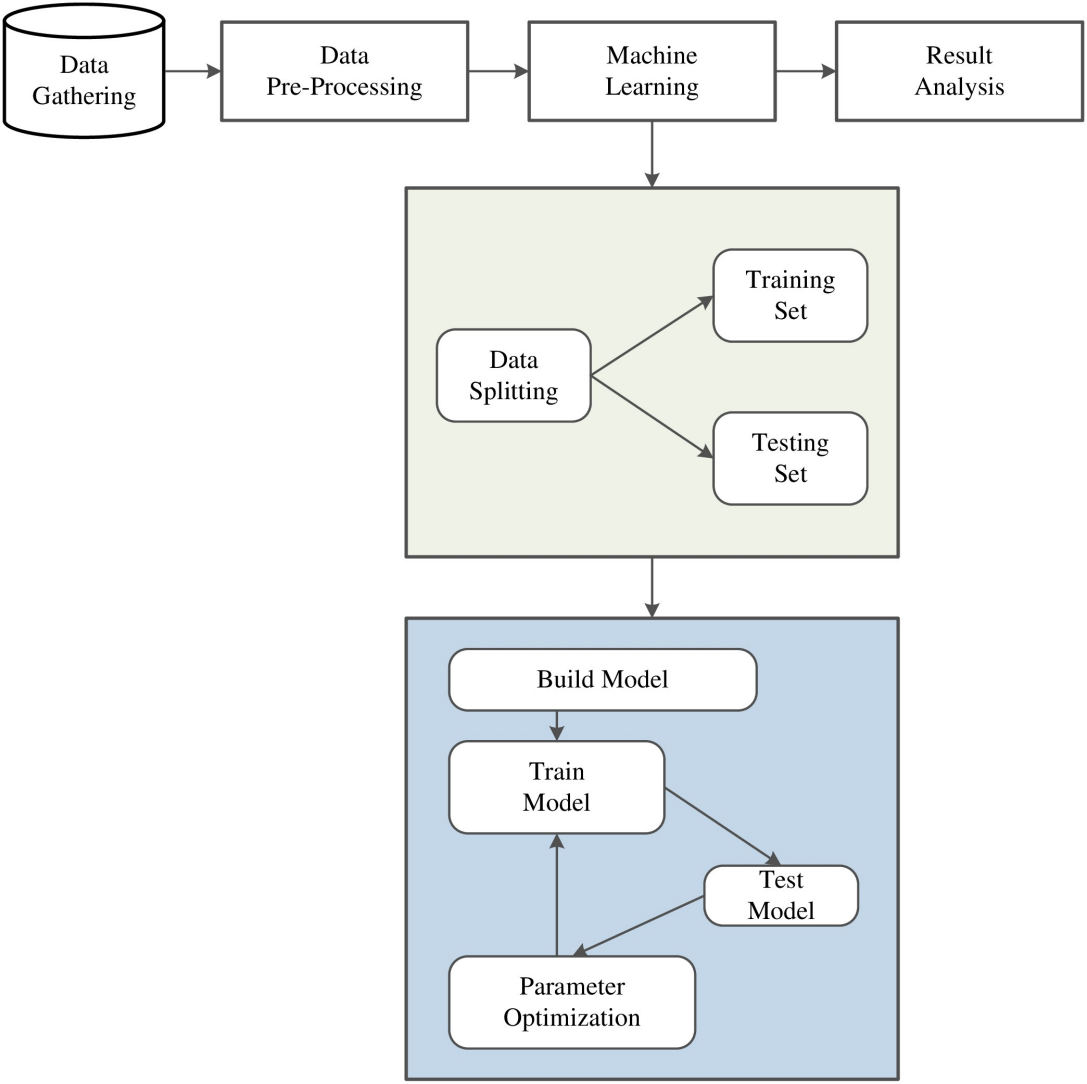
Framework diagram of performance prediction model.

The EGWO-MLP prediction model aims to predict student achievement and then determine the essential variables affecting educational success or failure. The hidden layer structure characteristics and dynamic weight parameter adjustment make it more suitable for predicting student final achievement. The prediction of student achievement can be expressed in Fig 3.

**Fig 4:**
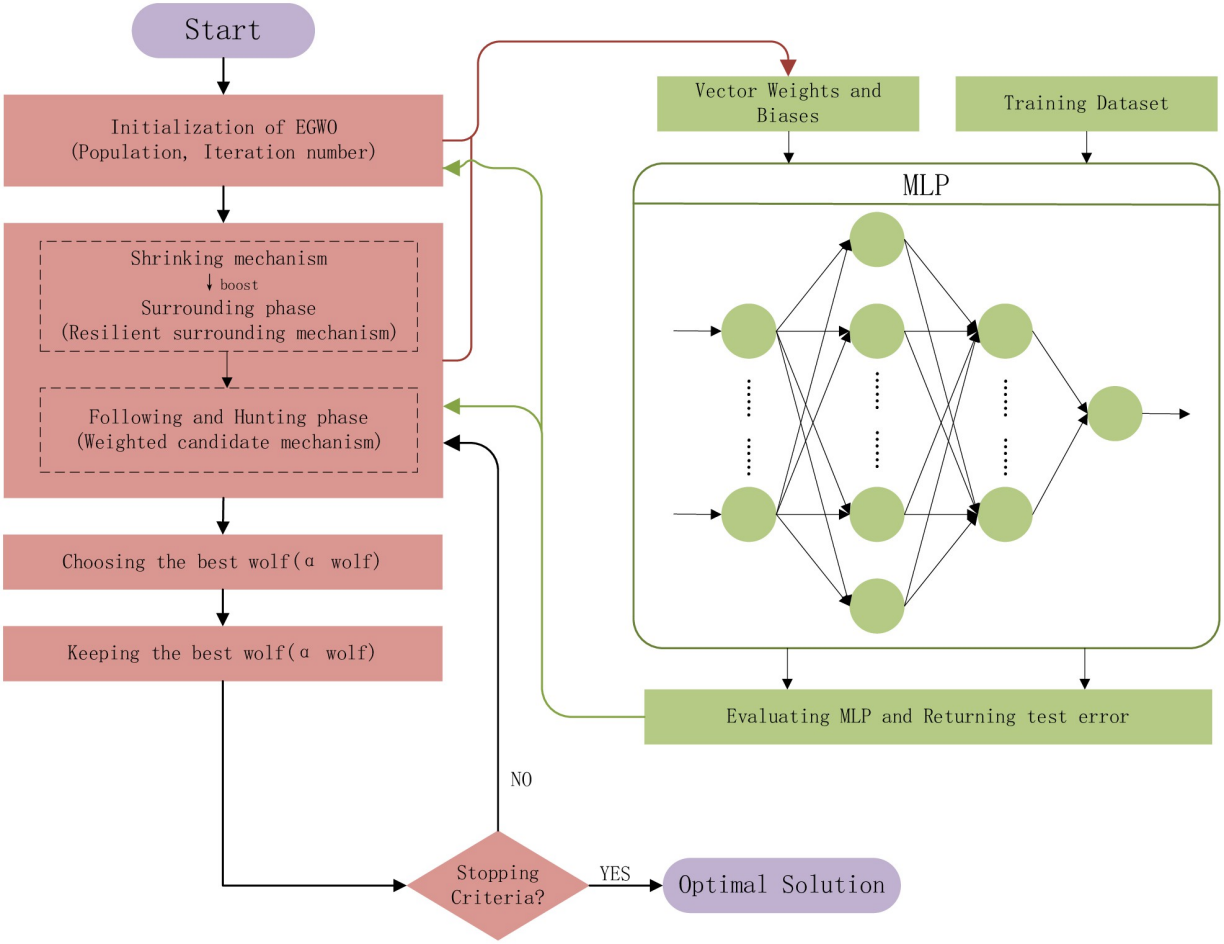
Process of Elastic Grey Wolf Optimization algorithm (EGWO) training MLP.

**Fig 5:**
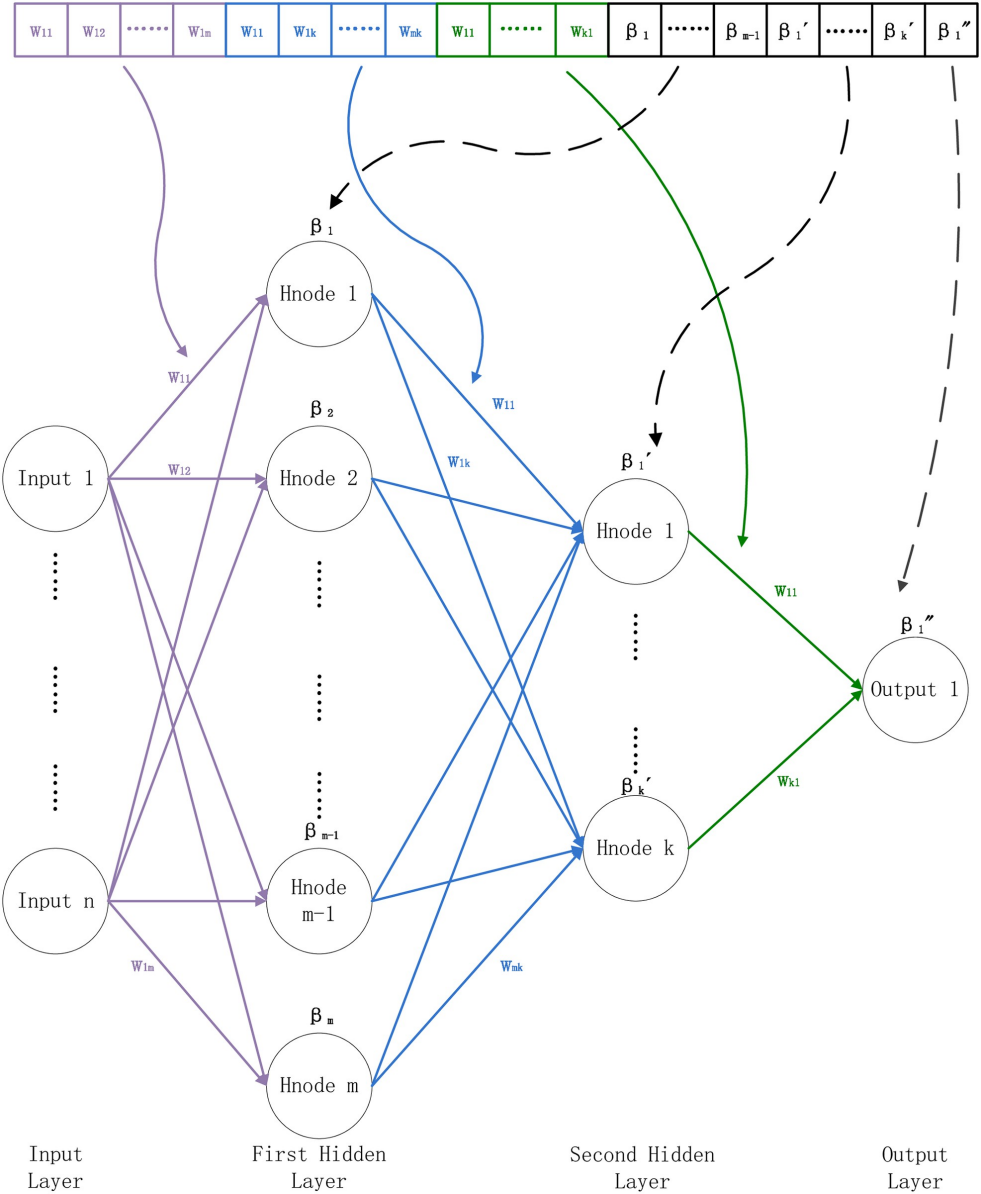
Weights and biases assignments of the Multi-Layer Perceptron (MLP) with two layers.

The model constructions contain input, operation, and output. The process includes normalization processing, determination of input, output and hidden units, training parameters setting, network model creation, calling of activation function, etc. The output is predicting outcomes. If the test sample’s output meets the training sample’s expectation, the learning ends. If it does not meet the expectation of the training sample, it learns again and adjusts the threshold until meeting the termination conditions. The whole process of EGWO training MLP is shown in Fig 4, and weights and biases assignments are presented in Fig 5.

## Experimental dataset and environment

### Student data

**Fig 6:**
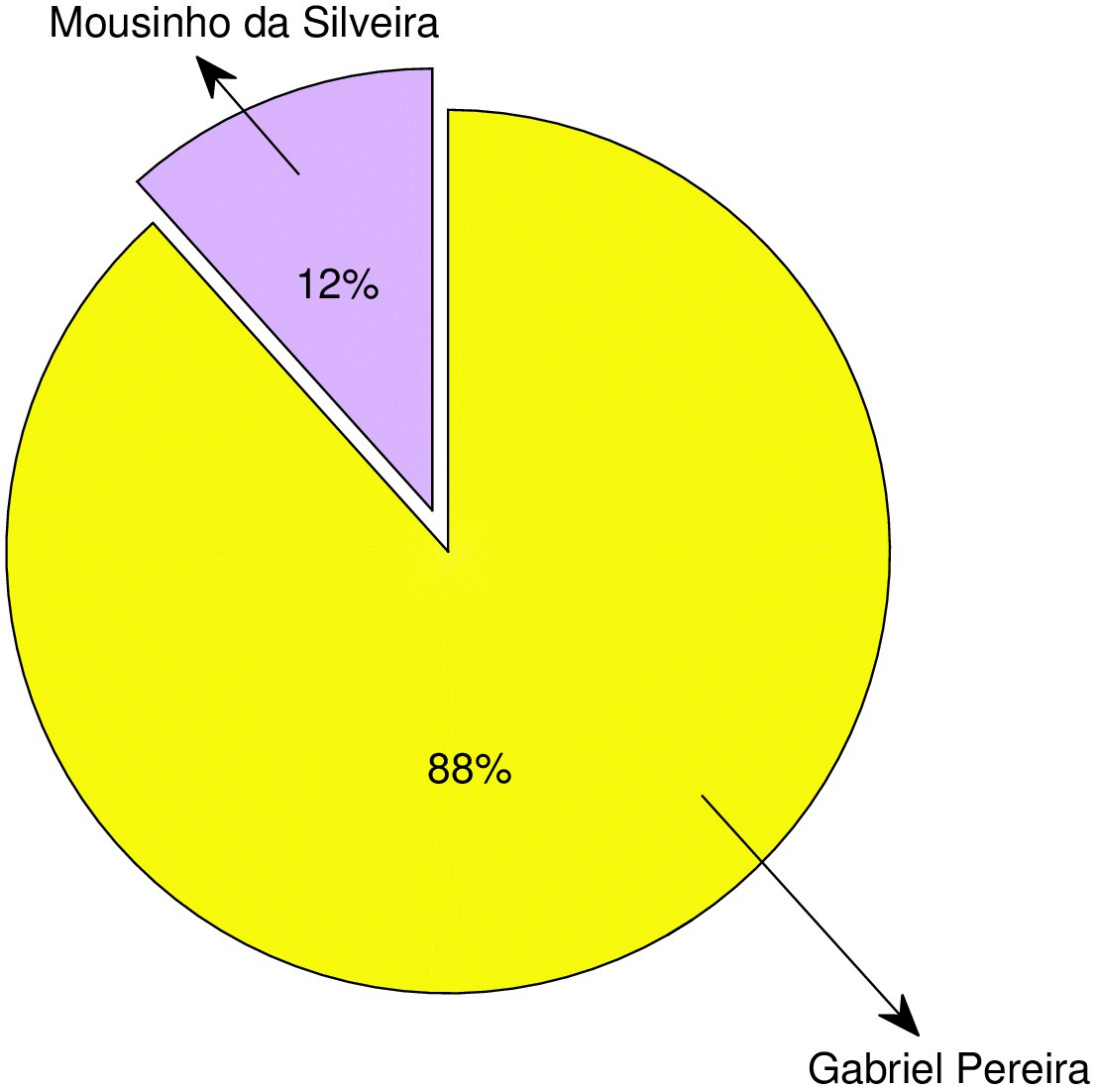
Proportions of two Portuguese schools.

In this study, we will analyze recent real world data from two Portuguese secondary schools to train and verify the prediction model obtained from the student performance University of California (UCI) Machine Learning Repository dataset (https://archive.ics.uci.edu/ml/datasets/Student+Performance). This dataset was collected through reports and questionnaires on students’ performance in secondary education in two Portuguese schools. The two schools’ proportions of the UCI dataset are shown in Fig 6. The data attributes include student achievement, demographic, social, and school-related characteristics and provide two data sets on the performance of two different subjects: Mathematics (Mat.) and Portuguese (Por.) [68]. The dataset contains thirty attributes, and they are shown in Table 1. The second column of the Table 1 shows the names of the thirty attributes and the third column of the Table 1 describes each attribute. The thirty attributes are the input of the prediction model. And for this paper, we set 80% training data and 20% testing data.

**Table 1:** Attribute information for student performance data set.

	Attribute Information	Description
1	school	student’s school (binary: GP - Gabriel Pereira or MS - Mousinho da Silveira)
2	sex	student’s sex (binary: F - female or M - male)
3	age	student’s age (numeric: from 15 to 22)
4	address	student’s home address type (binary: U - urban or R - rural)
5	famsize	family size (binary: LE3 - less or equal to 3 or GT3 - greater than 3)
6	Pstatus	parent’s cohabitation status (binary: T - living together or A - apart)
7	Medu	mothAttributeer’s education (numeric: 0 - none, 1 - primary education (4th grade), 2- 5th to 9th grade, 3 -secondary education or 4-higher education)
8	Fedu	father’s education (numeric: 0 - none, 1 - primary education (4th grade), 2 - 5th to 9th grade, 3 -secondary education or 4 - higher education)
9	Mjob	mother’s job (nominal: teacher, health care related, civil services)
10	Fjob	father’s job (nominal: teacher, health care related, civil services)
11	reason	reason to choose this school (nominal: close to home, school reputation, course preference or other)
12	guardian	student’s guardian (nominal: mother, father or other)
13	traveltime	home to school travel time (numeric: 1 -<15 min., 2 - 15 to 30 min., 3 - 30 min. to 1 hour, or 4 - >1 hour)
14	studytime	weekly study time (numeric: 1 -<2 hours, 2 - 2 to 5 hours, 3 - 5 to 10 hours, or 4 - >10 hours)
15	failures	number of past class failures (numeric: n if 1 ≤ n <3, else 4)
16	schoolsup	extra educational support (binary: yes or no)
17	famsup	family educational support (binary: yes or no)
18	paid	extra paid classes within the course subject (Mathematics or Portuguese) (binary: yes or no)
19	activities	extra-curricular activities (binary: yes or no)
20	nursery	attended nursery school (binary: yes or no)
21	higher	wants to take higher education (binary: yes or no)
22	internet	Internet access at home (binary: yes or no)
23	romantic	with a romantic relationship (binary: yes or no)
24	famrel	quality of family relationships (numeric: from 1 - very bad to 5 - excellent)
25	freetime	free time after school (numeric: from 1 - very low to 5 - very high)
26	goout	going out with friends (numeric: from 1 - very low to 5 - very high)
27	Dalc	workday alcohol consumption (numeric: from 1 - very low to 5 - very high)
28	Walc	weekend alcohol consumption (numeric: from 1 - very low to 5 - very high)
29	health	current health status (numeric: from 1 - very bad to 5 - very good)
30	absences	number of school absences (numeric: from 0 to 93)

### Prediction model (EGWO-MLP) parameters setting

The EGWO-MLP prediction model contains an input layer, two hidden layers, and an output layer. The input layer of the EGWO-MLP selects 30 attributes from the student performance UCI dataset, including family features and personal characteristics as the input nodes. The hidden layer sets to 2, the first hidden layer obtains (2 × *numbers of input* + 1) nodes, and the second hidden layer owns two. In the prediction model of this paper, 30 factors that affect students are used as the input of the model, 2 hidden layers, G1 and G2, are used as hidden nodes, and finally, the student’s grades are used as the output of the model.

### Experimental environment setting

The experimental environment adopts MATLAB, an advanced technical computing language and interactive environment integrating numerical analysis, data visualization, matrix calculation, and non-linear dynamic modeling. The experiment codes in Matlab R2015b environment under the Windows 10 operating system, all simulations run on the computer with Intel Core(TM) i3-6100 CPU @ 3.70GHz, and its memory is 8G. Twenty runs for each working accesses the predictive performances. The population and max iteration are 10 and 300, and the comparison algorithm’s parameter settings are shown in Table 2.

**Table 2:** List of parameter setting used algorithms-MLP.

Algorithms	Parameter	Value
EGWO	*a* (control parameter)	liner from 2 to 0
GWO	*a* (control parameter)	liner from 2 to 0
GWO	*a* (control parameter)	liner from 2 to 0
PSO	cognitive component	2
	social component	2
BA	Null	Null
DE	scale factor primary	0.6
	scale factor secondary	0.5
	scale factor secondary	0.3
	crossover rate	0.8
SCA	Null	Null
GA	CrossPercent	70%
	MutatPercent	20%
	ElitPercent	10%

### Criteria for evaluating performance

The **training error** is the error between the value predicted by the model and the actual value in the training set. The Mean Square Error (MSE) is the training error for the training part. MSE is the distinction between the actual and the predicted value obtained by the training algorithm [69, 70], which is widely used as a criterion [71]. And MSE is computed by Eq (28).

$$\begin{array}{c} MSE=\sum_{i=1}^{m}{\left(o_{i}^{k}-d_{i}^{k}\right)}^{2} \end{array}$$ (28)

where *m* is the output numbers, $d_{i}^{k}$ utilize *k*_*th*_ training sample to get the required output value of the *i*_*th*_ input, and $o_{i}^{k}$ is actual value of the *k*_*th*_ training sample. To ensure the fairness and effectiveness of the experiment, the average MSE ($\bar{MSE}$) for all training samples is computed by Eq (29).

$$\begin{array}{c} \bar{MSE}=\sum_{k=1}^{s}\frac{\sum_{i=1}^{m}{\left(o_{i}^{k}-d_{i}^{k}\right)}^{2}}{s} \end{array}$$ (29)

where *s* is the number of training samples, the training of an MLP consists of multiple variables and functions, where $\bar{MSE}$ for the EGWO algorithm is calculated by the Eq (30).

$$\begin{array}{c} minimize:F\left(\vec{V}\right)=\bar{MSE} \end{array}$$ (30)

The **test error** is the average error of the model on the test set, which measures the model’s generalization ability. In practice, the test error should be as small as possible.

## Experimental results and discussion

**Fig 7:**
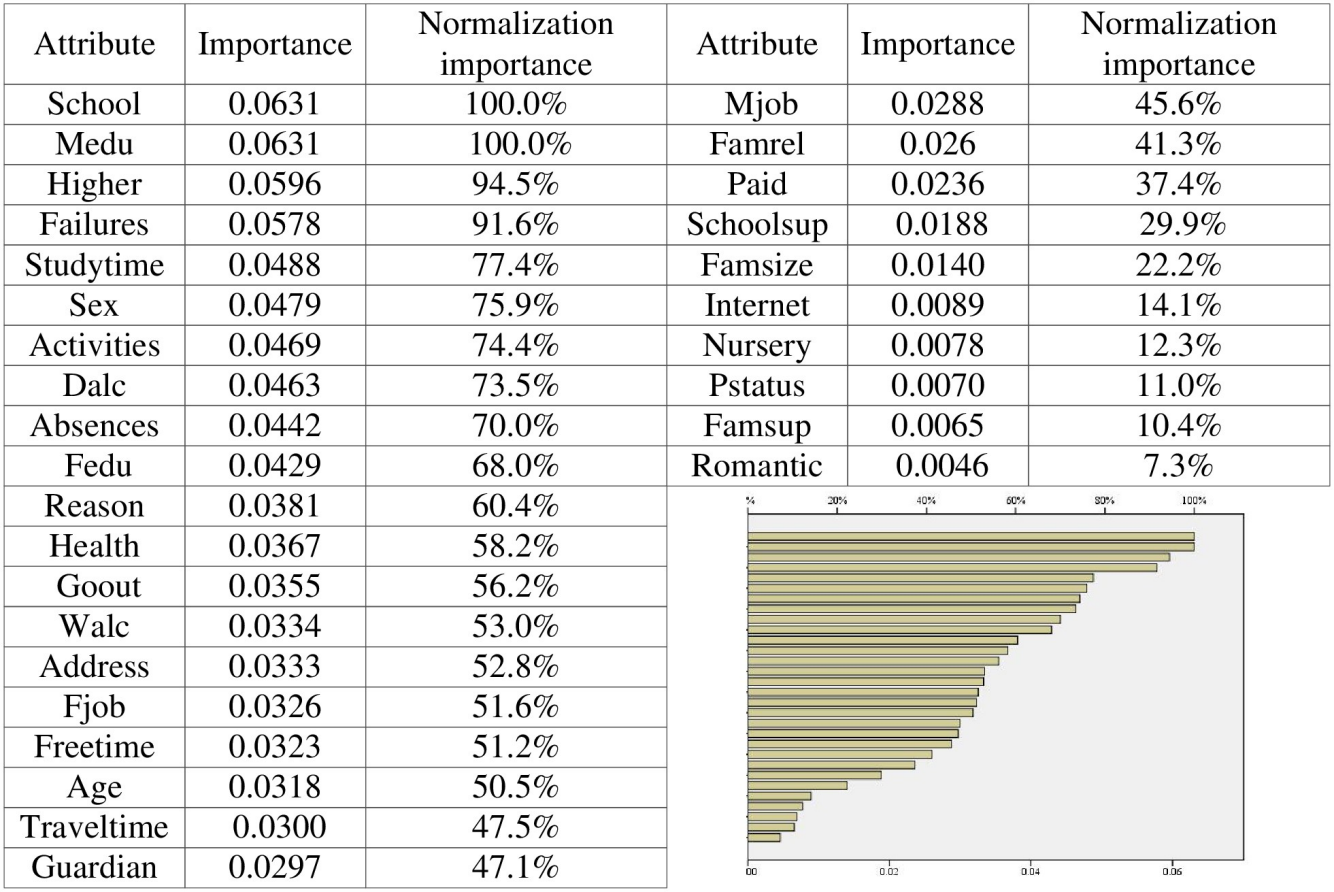
Results of variable importance.

**Fig 8:**
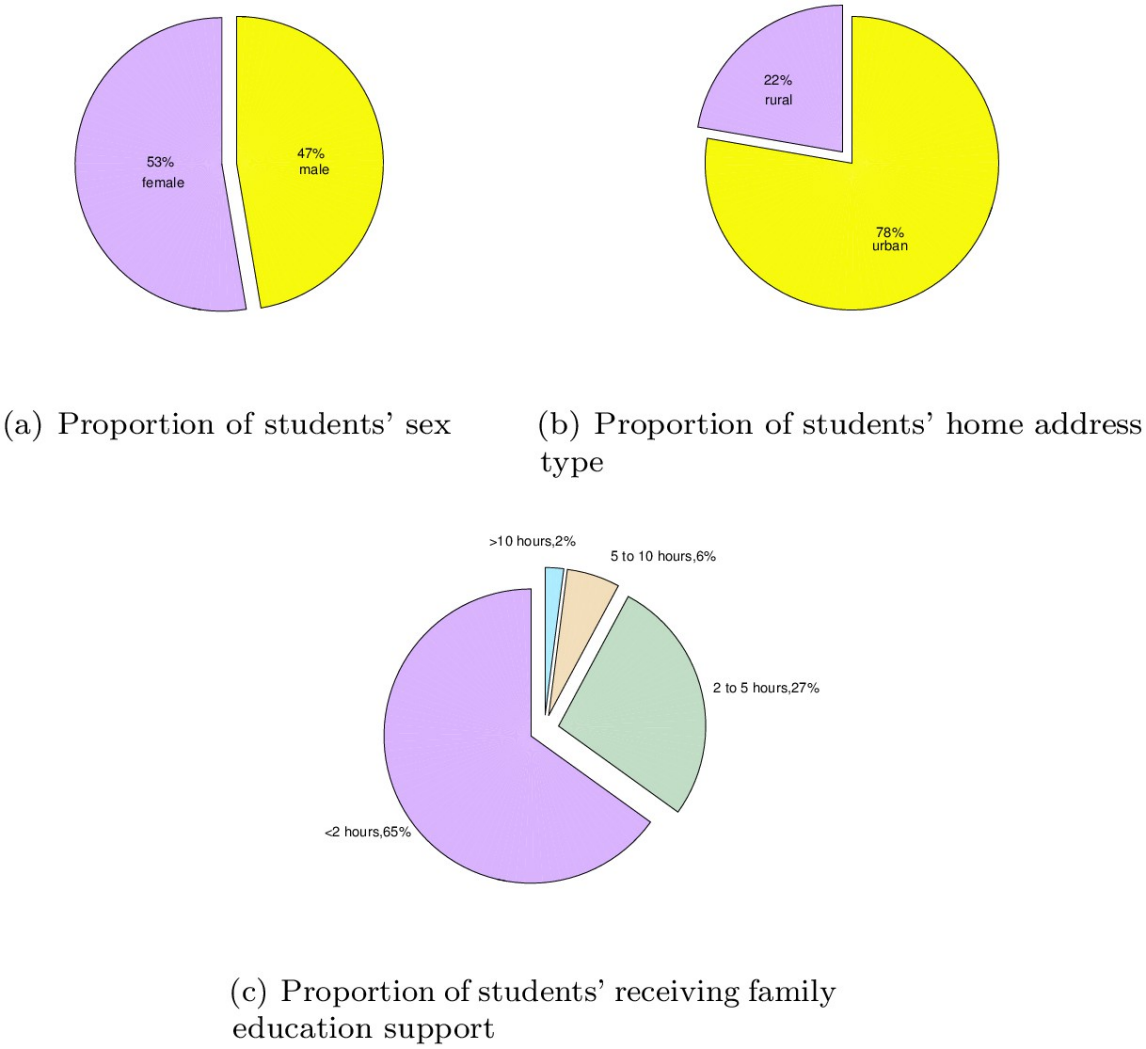
Students’ own various characteristics. (a) Proportion of students’ sex, (b) Proportion of students’ home address type, (c) Proportion of students’ receiving family education support.

**Fig 9:**
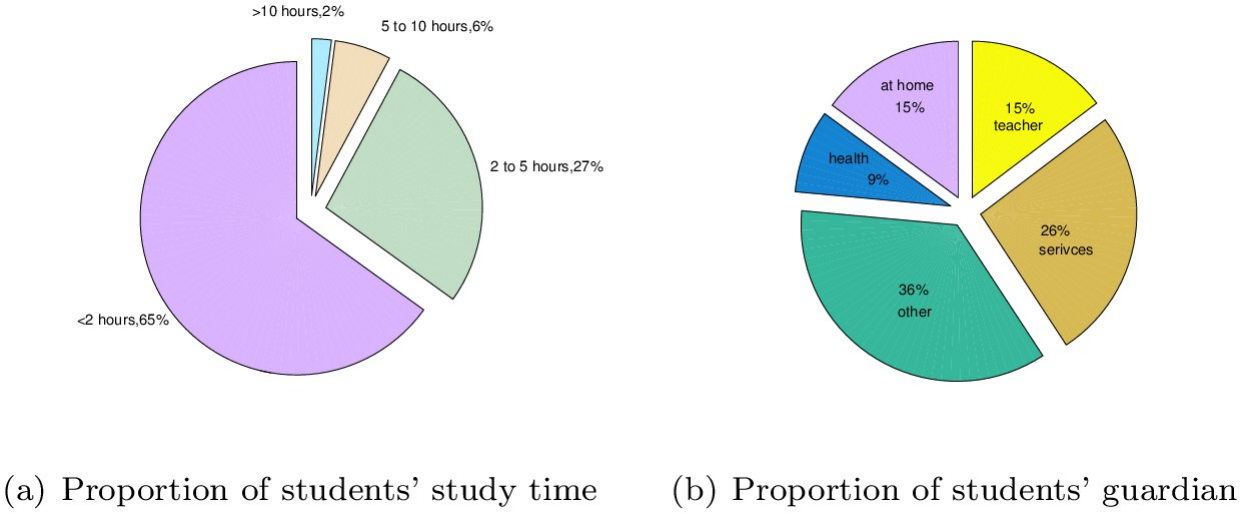
Study time and family guardian of the students. (a) Proportion of students’ study time, (b) Proportion of students’ guardian.

**Fig 10:**
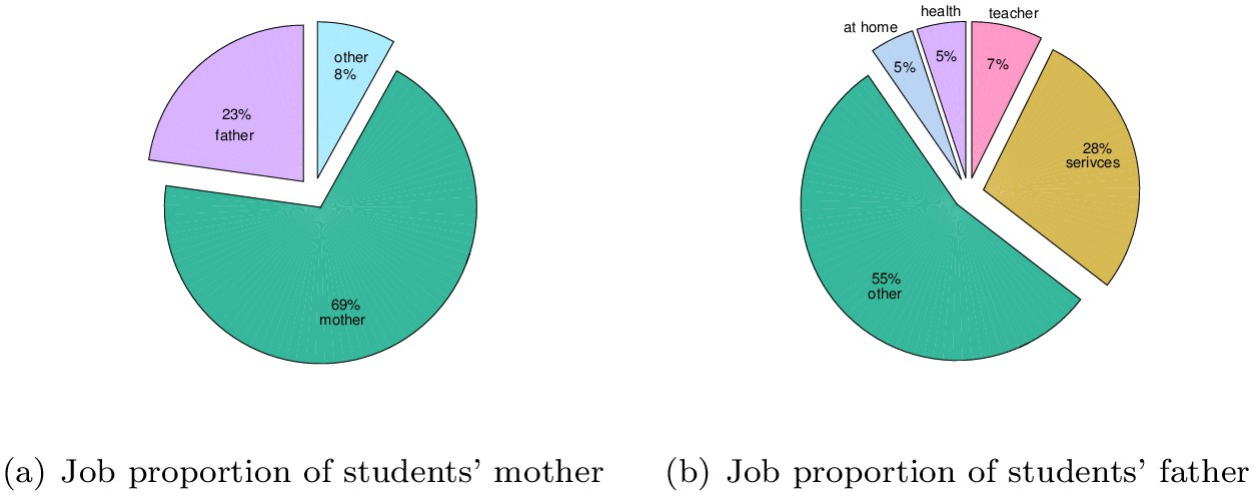
Job of the students’ parents. (a) Job proportion of students’ mother, (b) Job proportion of students’ father.

To further analyze the variables that affect student achievement, SPSS software is used to analyze the dataset to obtain the variable importance, and the results are shown in Fig 7. According to the results, it can be seen that the importance of the selected 30 variables to the final output results is different. According to the analysis of the UCI dataset, the proportion of girls in the survey reach 53%, as shown in Fig 8(a). As seen from Fig 8(b), most students’ home addresses are in cities. As shown in Fig 8(c), among the surveyed students, there are relatively more students with family support education. The time students spend learning also determines their degree of knowledge acquisition. According to Fig 9(a), most students study for less than two hours every week. For students, guardians have a direct impact on students’ living environment and then on students’ learning environment. It can be seen from Fig 9(b) that the guardians of most students are mothers, accounting for 69%. Job distribution of students’ parents has displayed in Fig 10, and other jobs account for a large proportion. In addition, service workers account for a large proportion, reaching 26% for father and 26% for mother. And the different importance of variables is selected to discuss the results, such as sex, home address style, family educational support, study time, and guardian and parents’ job.

Based on the above factors, we conclude that EGWO-MLP is a student achievement prediction model, and 30 factors, including the above ones, are input to forecast student achievement. To verify the optimization superiority of the EGWO algorithm, it is compared with other algorithms, including Advanced Grey Wolf Optimization algorithm (AGWO) [19], Particle Swarm Optimization (PSO) [72], Genetic Algorithm (GA) [73], Bat Algorithm (BA) [74], Differential Evolution (DE) [75], and Sine cosine algorithm (SCA) [76]. The model for various algorithms can be named AGWO-MLP, PSO-MLP, GA-MLP, BA-MLP, DE-MLP, and SCA-MLP models. Therefore, the EGWO-MLP model is compared with basic GWO-MLP, AGWO-MLP, PSO-MLP, GA-MLP, BA-MLP, DE-MLP, and SCA-MLP models. At the end of this process, the evaluated test set contains the entire data set, although ten various algorithms of the same MLP model create prediction results. As shown in the statistical test results, the “Do Not Test” occurs for a comparison when no significant difference is found between the two rank sums that enclose that comparison.

### Discussion 1: Mathematics (Mat.)

For the Mathematics (Mat.) subject achievement prediction, it can be seen from Tables 3 and 4, the EGWO-MLP model among the swarm intelligence optimization algorithms can obtain the best results. It is worse than the evolutionary algorithms. However, according to the standard deviation, EGWO-MLP can get the smallest standard deviation (std) in the training error rate. Due to the strong exploration and local stagnation avoidance of the EGWO, it can effectively feedback weights and biases to predict student achievement compared with the basic GWO-MLP. To further verify the performance of the EGWO-MLP model and the difference from other algorithms-MLP models, we choose the ANOVA on the RANKS test. And the experimental results are shown in Table 5, demonstrating that EGWO-MLP is superior to AGWO-MLP, GWO-MLP, and GA-MLP models. In general, EGWO-MLP has a significant advantage in predicting student achievement.

**Table 3:** The training error of the Mathematics (Mat.) subject achievement prediction.

Run No.	EGWO-MLP	AGWO-MLP	GWO-MLP	PSO-MLP	BA-MLP	DE-MLP	SCA-MLP	GA-MLP
1	0.2276	0.2335	0.1978	0.2234	0.2335	0.2125	0.2284	0.2336
2	0.2297	0.2336	0.1977	0.2318	0.2318	0.1987	0.2263	1.1141
3	0.2300	0.2435	0.1967	0.2335	0.2335	0.2155	0.2256	0.2335
4	0.2272	0.2336	0.2097	0.2335	0.2336	0.2170	0.2258	0.2328
5	0.2297	0.2335	0.1993	0.2284	0.2335	0.2099	0.2264	0.2336
6	0.2280	0.2335	0.2011	0.2301	0.2332	0.2152	0.2271	0.2332
7	0.2283	0.2334	0.1961	0.2334	0.2328	0.2224	0.2295	0.2313
8	0.2292	0.2331	0.1928	0.2312	0.2335	0.2089	0.2263	0.2342
9	0.2300	0.2325	0.1876	0.2293	0.2336	0.2212	0.2289	0.2334
10	0.2256	0.2336	0.1936	0.2336	0.2334	0.2148	0.2253	0.2337
11	0.2267	0.2415	0.1970	0.2325	0.2255	0.2201	0.2300	0.2364
12	0.2248	0.2335	0.2202	0.2256	0.2335	0.2219	0.2251	0.2336
13	0.2290	0.2334	0.2040	0.2334	0.2336	0.2167	0.2271	0.2335
14	0.2264	0.2335	0.2044	0.2300	0.2324	0.2125	0.2259	0.2335
15	0.2281	0.2335	0.2014	0.2319	0.2311	0.2216	0.2272	0.2335
16	0.2280	0.2336	0.1968	0.2328	0.2332	0.2180	0.2262	0.2344
17	0.2258	0.2328	0.2045	0.2272	0.2335	0.2004	0.2268	1.2090
18	0.2235	0.2315	0.1929	0.2291	0.2334	0.2190	0.2289	0.2336
19	0.2282	0.2336	0.2010	0.2255	0.2316	0.2213	0.2264	0.2335
20	0.2267	0.2336	0.2050	0.2255	0.2329	0.2178	0.2250	0.2335
Average	0.2276	0.2342	0.2000	0.2301	0.2327	0.2153	0.2269	0.3264
STD	**0.0018**	0.0029	0.0070	0.0032	0.0018	0.0067	0.0015	0.2860

**Table 4:** The test error of the Mathematics (Mat.) subject achievement prediction.

Run No.	EGWO-MLP	AGWO-MLP	GWO-MLP	PSO-MLP	BA-MLP	DE-MLP	SCA-MLP	GA-MLP
1	0.8203	0.5607	0.5106	11.1971	19.4159	1.7919	5.6826	0.5563
2	1.2157	17.3616	7.1759	0.5794	19.3343	0.5455	2.7689	0.4369
3	19.4193	0.8438	2.5727	15.3038	1.2488	1.2666	19.4200	0.2529
4	3.3607	0.4689	17.4418	19.3830	14.8622	0.5782	3.9617	0.5794
5	5.0453	0.5728	3.7694	14.9889	0.5795	7.2811	2.2985	2.4488
6	1.4397	0.5643	6.4064	16.4883	1.2694	19.4168	0.5794	18.9813
7	0.7536	0.5378	2.8849	19.4057	14.7706	0.5795	7.0829	10.3603
8	1.3838	0.5428	5.7407	8.2803	19.4205	0.5792	19.2652	17.9100
9	1.7541	12.3752	13.7732	0.5791	0.5795	17.6510	7.1295	0.6903
10	11.6022	12.5526	0.5624	0.5742	19.4205	0.5793	1.4137	11.2883
11	0.7447	19.4193	5.5138	0.8799	0.5616	8.2135	18.2744	19.1053
12	9.7731	0.7597	0.8398	17.5704	0.5795	19.4196	1.3015	19.4183
13	0.7900	16.9240	0.4161	3.7852	0.5795	2.2360	18.4916	0.6752
14	12.6490	0.5728	2.1260	0.5625	12.2708	6.4231	0.7156	6.9284
15	0.5795	0.6662	5.2246	1.3556	10.4316	1.0817	0.4074	11.4678
16	13.3296	0.5762	0.5743	19.1369	19.4197	0.6789	19.4198	0.5780
17	13.6841	19.3461	1.5619	0.8434	19.4205	0.5781	17.0050	19.4205
18	0.6659	0.8462	3.4386	2.9293	0.5795	0.5551	19.4180	3.0454
19	0.5650	1.6344	6.0814	15.4490	3.8960	2.5732	1.7938	0.5669
20	5.1548	2.6658	19.0786	0.5616	9.5842	0.5355	0.9438	19.3427
Average	5.2365	5.4896	5.2847	8.4927	9.4112	**4.6282**	8.3687	8.2026
STD	5.8899	7.4759	5.4736	7.8671	8.2673	6.5681	8.0707	8.1460

**Table 5:** Results for RM ANOVA on RANKS of the Mathematics (Mat.) subject achievement prediction.

EGWO-MLP	Diff of Ranks	q	P	P<0.050
vs AGWO-MLP	1210.5	5.842	<0.001	**Yes**
vs GWO-MLP	1144.5	5.524	0.002	**Yes**
vs PSO-MLP	416.5	2.01	0.849	Do Not Test
vs BA-MLP	915	4.416	0.038	**Yes**
vs DE-MLP	810.5	3.912	0.104	No
vs SCA-MLP	133	0.642	1	Do Not Test
vs GA-MLP	1326	6.4	<0.001	**Yes**

### Discussion 2: Portuguese (Por.)

For the Portuguese (Por.) subject, as shown in Tables 6 and 7, EGWO-MLP can outperform most models based on swarm intelligence optimization algorithms during model training. However, due to the unique evolutionary characteristics of evolutionary algorithms, it is difficult for EWGO-MLP to surpass its optimization model. For example, the remarkable difference strategy of DE makes it enhance the exploration ability and avoid local stagnation in the optimization process. During the testing process, it is difficult for the compared models to achieve stable model optimization, and EGWO-MLP can obtain the lowest test error and standard deviation. The experimental results of statistical tests are shown in Table 8. The experimental results show that its EGWO-MLP can outperform most of the compared models and has strong stability.

**Table 6:** The training error for the Portuguese (Por.) subject achievement prediction.

Run No.	EGWO-MLP	AGWO-MLP	GWO-MLP	PSO-MLP	BA-MLP	DE-MLP	SCA-MLP	GA-MLP
1	0.0963	0.1047	0.0609	0.1005	0.1087	0.0672	0.0939	0.1019
2	0.0990	0.1044	0.0654	0.1857	0.1041	0.0784	0.0900	0.1049
3	0.0968	0.1018	0.0589	0.0986	0.1000	0.0797	0.0952	0.1100
4	0.0982	0.1014	0.0560	0.1013	0.1020	0.0879	0.0951	0.1094
5	0.0990	0.1094	0.1013	0.1008	0.1021	0.0820	0.0949	0.1128
6	0.0992	0.1098	0.0602	0.0985	0.0997	0.0826	0.0945	0.4532
7	0.0985	0.1100	0.0653	0.0857	0.1014	0.0959	0.0972	0.0987
8	0.0978	0.1019	0.0568	0.0991	0.1035	0.0836	0.0914	0.1094
9	0.0968	0.1014	0.0693	0.0909	0.1018	0.0742	0.0937	0.1081
10	0.0940	0.1097	0.0657	0.1001	0.1064	0.0739	0.0968	0.1067
11	0.1001	0.1097	0.0650	0.0795	0.1098	0.0833	0.0946	0.1098
12	0.1013	0.1041	0.0672	0.0943	0.1016	0.0916	0.0948	0.1002
13	0.0950	0.1099	0.0715	0.0963	0.1076	0.0867	0.0958	0.1057
14	0.1002	0.1046	0.0555	0.1010	0.1002	0.0867	0.0935	0.1022
15	0.0978	0.1045	0.0572	0.1001	0.1011	0.0817	0.0942	0.1009
16	0.1013	0.1071	0.0995	0.0848	0.1098	0.0805	0.0987	0.1014
17	0.0977	0.1050	0.0739	0.1013	0.1006	0.0864	0.0971	0.0932
18	0.0996	0.1094	0.0596	0.0940	0.1014	0.0738	0.0957	0.1019
19	0.0926	0.1085	0.0607	0.0960	0.1014	0.0767	0.0930	0.1084
20	0.1000	0.1039	0.0607	0.1062	0.0958	0.0780	0.0958	0.4351
Average	0.0981	0.1061	0.0665	0.1007	0.1029	0.0815	0.0948	0.1387
STD	0.0023	0.0032	0.0127	0.0210	0.0037	0.0067	0.0020	0.1046

**Table 7:** The test error for the Portuguese (Por.) subject achievement prediction.

Run No.	EGWO-MLP	AGWO-MLP	GWO-MLP	PSO-MLP	BA-MLP	DE-MLP	SCA-MLP	GA-MLP
1	0.5423	6.8999	0.6107	0.6261	0.6261	16.0867	11.6129	0.4291
2	1.9032	7.4782	0.7279	18.1323	18.4162	9.3749	18.4148	18.2308
3	0.6257	0.5905	1.5776	0.6794	3.8775	0.3586	18.3992	18.2124
4	0.7377	1.9999	4.7575	18.4162	0.6262	0.6256	0.5343	0.5586
5	1.2717	18.4155	0.6522	0.6209	18.3802	11.9379	4.9711	18.4155
6	0.6178	0.6199	14.9946	0.4471	13.6740	2.8972	12.2347	6.8621
7	1.5704	0.6454	12.5947	5.1917	18.4162	13.2596	18.3792	18.4071
8	0.6254	0.6015	0.3794	10.9449	0.6262	16.1895	11.9942	18.1568
9	0.6254	0.6158	18.4158	13.7330	18.3274	12.2983	5.4923	18.2699
10	0.7742	0.6134	1.4392	0.7011	16.9485	14.9986	0.6246	18.4001
11	0.7972	16.7027	7.6506	4.4963	15.3922	17.2430	18.4155	0.5740
12	0.6253	18.4162	7.8011	17.7680	0.6075	7.8527	2.2755	4.4038
13	1.3291	0.2718	18.0234	8.0490	18.3636	17.4994	14.9837	3.0688
14	0.6262	0.4925	4.5754	18.4158	0.6261	18.3946	0.9636	17.3330
15	0.9300	18.4080	0.6176	7.7548	1.2499	16.9969	1.4260	0.4256
16	0.6253	11.5751	17.8137	9.3616	16.5916	11.4802	3.1054	16.7099
17	1.2472	0.6224	17.9938	0.3884	0.5623	18.3807	11.7712	17.0762
18	0.6253	0.8996	5.4777	17.0106	0.5878	7.2656	0.6251	18.4075
19	2.7785	18.4159	0.5038	18.3411	18.4162	12.1422	10.1350	18.4134
20	0.6246	18.4081	7.6013	5.0331	0.6262	8.9482	3.1016	0.6213
Average	**0.9751**	7.1346	7.2104	8.8056	9.1471	11.7115	8.4730	11.6488
STD	**0.5703**	7.9517	6.9060	7.2305	8.4637	5.6728	6.8682	8.1324

**Table 8:** Results for RM ANOVA on RANKS of the Portuguese (Por.) subject achievement prediction.

EGWO-MLP	Diff of Ranks	q	P	P <0.050
vs AGWO-MLP	1117	5.391	0.003	**Yes**
vs GWO-MLP	1204	5.811	<0.001	**Yes**
vs PSO-MLP	34.5	0.167	1	Do Not Test
vs BA-MLP	748	3.61	0.174	No
vs DE-MLP	956	4.614	0.024	**Yes**
vs SCA-MLP	425.5	2.054	0.834	Do Not Test
vs GA-MLP	982	4.739	0.018	**Yes**

To sum up, this section selects two subjects (Mathematics (Mat.) and Portuguese (Por.)) to train and test the model. The experimental results show that EGWO-MLP is better than the selected swarm intelligence optimization algorithm model. It is difficult to outperform the model trained by the typical evolutionary algorithm in the training process in terms of the unique evolution strategy. However, the testing process shows that EGWO-MLP is more stable and effective and can outperform the compared models. The shrinking, resilient surrounding and weighted candidate mechanisms can decide the wolf position to update the step direction and length. The operation is instrumental in accelerating the convergence speed, avoiding local stagnation, and balancing exploration and exploitation. The above advantages ensure that EGWO can effectively optimize the weights and biases of MLP to drive the EGWO-MLP model to solve high-dimensional complex problems and analyze a large amount of data. To prove the experiment’s validity, the experimental results are statistically analyzed, demonstrating that EGWO-MLP model is effective in dealing with the problem of student achievement prediction. Through the analysis of the above EGWO-MLP model, the selected thirty inputs are conducive to predicting student achievement. For the thirty variables, the weight of their importance is copied through EGWO to avoid the effect of weight assignment of objective reasons on the final prediction results.

## Suggestions

Through the model construction in the above chapters and the analysis and discussion of the experimental results, the enlightenment to promote student performance and teaching effectiveness are shown in the following aspects:

Firstly, with the rapid development of modern information technology, communication technology, and computer technology, database application’s scope, depth and scale is expanding. Big data mining and analysis can also benefit educational institutions at all levels. Currently, the use of data stored in schools’ management systems is mainly in a relatively primary stage. Generally, only simple queries and statistical tables are provided in the system, while a large amount of information affecting students’ learning is not accessible. Data mining and analysis through the EGWO-MLP model can make full use of the obtained data to reveal the correlation between student performance and family and individual. And school factors, more accurately, can provide the basis for school decision-makers and help them more comprehensively monitor and regulate the factors affecting teaching quality to ensure the quality of education.

Secondly, by mining the main factors affecting student achievement in a subject, the EGWO-MLP model is generated. Through the analysis of the characteristics of learners, we can understand the learning environment, cognitive factors, and learning ability of different learning individuals. On this basis, teachers can provide personalized teaching content and method according to group differences and learners’ characteristics, which can give the basis for teachers to adjust teaching strategies according to students’ aptitudes. This method can be applied to other subjects so that students can maintain a good learning state and improve the overall learning effect.

Thirdly, the EGWO-MLP model proposed in this paper can predict and warn students, which is conducive to assisting teachers in managing and helping students. Simultaneously, the prediction results can enhance self-learning awareness of students and improve the teaching quality of teachers. The current study can help teachers predict student achievement, reflect on teaching performance, and provide technical analysis strategies and management recommendations for high-quality training of teachers. And it can prevent teachers from selectively ignoring students with a poor foundation, resulting in the unfairness of the teaching process.

Fourthly, during the epidemic period of COVID-19, online teaching is more extensive, making it difficult to ensure students’ performance and effectively give guidance in study or training for students. Meanwhile, online learning achievement prediction mainly relies on structured data, which is difficult to profoundly and accurately mine learners’ states, emotions, and other information, affecting prediction accuracy. Therefore, inspired by this paper, swarm intelligence technology is combined with neural networks to improve prediction accuracy and education quality.

## Conclusion and future work

Under the pandemic trend of COVID-19, due to the change in curriculum arrangement and teachers’ teaching methods, student academic achievement and performance have become the focus of education. To effectively manage students’ learning factors and efficiently guide students in their learning, a direct and effective way is needed to predict student performance. The improvement of student performance and ability is a critical issue in education. The analysis of family features and personal characteristics on student achievement and performance finds that the case of student achievement prediction belongs to non-linear, high-dimensional, and complex practical problems. The existing NFL theory-based prediction methods fail to predict student achievement and performance, for they cannot fully cover influencing factors. To more accurately predict student performance, this paper builds a prediction model based on MLP.

MLP is a method applied to solve high-dimensional complex problems, and it has been applied to predict student achievement in precious research and educational practice. Since the MLP tends to fall into local stagnation, it is challenging to obtain the optimal solution during the optimization process, and finally obtain better optimization results. Therefore, introducing a swarm intelligence optimization algorithm optimizes the weights and biases of the MLP.

To solve the above problems and obtain better experimental results, this paper proposes an Elastic Grey Wolf Optimization algorithm (EGWO) variant of the grey wolf optimization algorithm. EGWO integrates with MLP to optimize the weights and biases to predict student achievement effectively. The **contribution** of the above can be summarized as follows:

- Shrinking and resilient surrounding mechanisms compute the positions of the *α*, *β*, and *δ* wolves to enhance exploration.
- Due to the introduction of the weighted candidate mechanism, the hunting operation is not limited to the *α* wolf. The occurrence of candidate wolves avoids local stagnation.
- The proposal of the EGWO algorithm optimizes the weights and biases of the MLP to obtain an accurate prediction value.
- The EGWO-MLP model is proposed to predict student achievement with reduced test error. It can fully mine data information and make full use of data information for prediction.

To verify the superiority of the proposed EGWO-MLP model, the achievement predictions of Mathematics (Mat.) and Portuguese (Por.) by the model are compared with those by AGWO-MLP, PSO-MLP, GA-MLP, BA-MLP, DE-MLP, and SCA-MLP models. The evaluation criteria include training (MSE) and test errors. The experimental results show that EGWO-MLP model has less test error and standard deviation. It demonstrates that this EGWO-MLP model can effectively predict student achievement. The corresponding suggestions and countermeasures are put forward through the analysis of the experimental results.

Experiments show that artificial neural network has certain advantages in student performance prediction, which can effectively manage and cultivate students. However, some limitation influences the prediction accuracy, such as the amount of data and feature attributes in existence referencing the UCI data. In the future, Convolutional Neural Networks (CNN) and Long short-term memory (STLM) can be selected to predict student performance according to the timeline. At the same time, more effective swarm intelligence technology can be chosen to optimize the neural network structure and adjust parameters to improve prediction accuracy.
